# The Role of Fiber in the Treatment of Functional Gastrointestinal Disorders in Children

**DOI:** 10.3390/nu10111650

**Published:** 2018-11-03

**Authors:** Cara Hannah Axelrod, Miguel Saps

**Affiliations:** Division of Pediatric Gastroenterology, Hepatology, and Nutrition, University of Miami Miller School of Medicine, Miami, FL 33136, USA; msaps@med.miami.edu

**Keywords:** functional constipation, irritable bowel syndrome, abdominal pain, fiber, children

## Abstract

We reviewed the available evidence on the role of fiber in the treatment of Functional Constipation (FC) and Irritable Bowel Syndrome (IBS) in children. The vast majority of toddlers and preschoolers do not consume enough fiber. Two of the most common reasons for consultation to a pediatric gastroenterology practice include FC and IBS. The North American Society for Pediatric Gastroenterology, Hepatology, and Nutrition (NASPGHAN) and the European Society of Pediatric Gastroenterology, Hepatology, and Nutrition (ESPGHAN) guidelines state that the evidence does not support the use of fiber supplements in the treatment of FC in children, and the Rome IV criteria do not recommend an increase in fiber consumption, in children with IBS. Despite this, in general practice, it is commonly recommended that children who experience constipation and IBS to increase their fiber intake. We conducted a systematic review of the available evidence on the role of fiber in the treatment of FC and IBS in children. Thirteen full-text articles with a total of seven hundred and twenty-three pediatric participants were included in this review. Three clinical trials found positive effects of dietary fiber for the management of IBS. Nine out of ten trials found fiber to be either more effective than placebo, or just as effective as laxative treatment. Most studies on the use of fiber for the treatment of FC and IBS have shown its benefit. However, due to the heterogeneity in study design, length of treatment, outcome measures, and amount and type of fiber, we were unable to make a definitive recommendation supporting the use of fiber for the treatment of FC and IBS in children.

## 1. Introduction

The Rome IV criteria classifies functional gastrointestinal disorders (FGIDs) in the disorders of nausea and vomiting, disorders of defecation (functional constipation (FC) and functional non-retentive fecal incontinence) and functional abdominal pain disorders (irritable bowel syndrome (IBS), functional dyspepsia, abdominal migraine, and functional abdominal pain not otherwise specified) [1].

FGIDS in children is a public health problem. Approximately, one in sixteen school-age children are diagnosed with IBS [2], most of them have IBS with constipation (IBS-C) [3]. FC alone has a reported prevalence, ranging between 0.7% and 29.6%, with a world-pooled prevalence of 9.5% [4]. Hereditary or environmental factors may play a role in the development of these conditions. FGIDs are associated with anxiety and lower health-related quality of life in physical, social, emotional, and school functioning [5]. As a result, IBS and FC are among the most common reasons for consultation with pediatric gastroenterology [6].

The treatment of FGIDs includes changes in lifestyle, diet, and medications, when needed. Some of the most commonly recommended dietary changes for FC are to increase the ingestion of fluids and fiber. Most of the water in the intestines is not ingested but results from intestinal secretions. Water is reabsorbed in the small and large intestine [7] with a very small percentage of it present in the stools [8]. Thus, it is unlikely that with the exclusion of extreme situations of water deprivation and dehydration [9], an increase in the intake of fluids could have an effect on the consistency or frequency of the stools. The North American Society for Pediatric Gastroenterology, Hepatology, and Nutrition (NASPGHAN) and the European Society of Pediatric Gastroenterology, Hepatology, and Nutrition (ESPGHAN) published clinical guidelines/position papers that concluded that the available evidence does not support the use of fiber supplements in the treatment of FC [10]. Despite the lack of evidence for both interventions, pediatric gastroenterologists commonly recommend water and fiber for the treatment of FC [11]. A survey conducted among pediatric gastroenterologists from Europe and the US found that 86% and 81% of them recommended water and fiber, respectively, for the treatment of FC in children [12]. The use of fiber for the treatment of IBS is controversial. It is commonly recommended to make dietary changes for IBS, such as limiting fermentable oligosaccharides polysaccharides and polyols (FODMAPs) that are high in fiber as those may worsen IBS symptoms. Still, fiber is sometimes recommended for the treatment of IBS.

Fibers are categorized as soluble or insoluble. Soluble fibers are fermented in the colon whereas insoluble fibers are subject to limited fermentation. Soluble fiber absorbs water, forming a gel after digestion. The added water is thought to make the stool softer and easier to pass. Common sources of soluble fiber include oatmeal, barley, beans, lentils, and peas. Insoluble fibers have a bulking action and can only be fermented in the colon producing gas and worsening the IBS symptoms [13]. Insoluble fibers do not get broken down completely in the body, and help food pass more quickly through the intestines. Wheat bran, whole grains, and some vegetables, all contain insoluble fiber.

A large number of soluble and insoluble fiber sources that have been studied for different ailments include, Gum Acacia, Alginate, Apple Fiber, Bamboo Fiber, Carboxymethyl Cellulose, Corn Hull Fiber, Cottonseed Fiber, Galactooligosaccharides (GOS), Inulin/Oligofructose/Synthetic Short-Chain Fructooligosaccharides, Karaya Gum, Oat Hull (Insoluble Oat) Fiber, pea fiber, Polydextrose, Potato Fibers, Pullulan, Rice Bran Fiber, High Amylose Corn/Maize Starch (Resistant Starch 2), Retrograded Corn Starch (Resistant Starch 3), Resistant Wheat and Maize Starch (Resistant Starch 4), Soluble Corn Fiber, Soy Fiber, Sugar Beet Fiber, Sugar Cane Fiber, Wheat Fiber, Xanthan Gum, and Xylooligosaccharides (XOS) [14]. Only few of them have been studied for the management of FGIDs.

The outcomes of treatment of FC and IBS are suboptimal. One in four children with FC and IBS continue to experience symptoms through adulthood [15]. Approximately 40% of children that consult for FC continue to require treatment after five years [16]. Parents frequently do not follow all the recommendations provided [17]. Only 37% [18] of parents that consult pediatric gastroenterology for the treatment of FC comply with the recommendations for laxatives. This may result from the desire for natural treatments, fear of side effects or difficulty in following a plan that may be too complex and with multiple recommendations [19]. Thus, to enhance adherence and treatment success, it is important to ensure that the recommendations given to the parents are simple and based on the best evidence [20].

We conducted a systematic review of the available evidence on the role of fiber, in the treatment of FC and IBS in children.

## 2. Materials and Methods

PubMed/Medline and Scopus were searched from initiation through 23 August, 2018. To find evidence for fiber therapy in children, basic search terms were inputted: “children”, “childhood”, “pediatrics”, “fiber”, “recurrent abdominal pain (RAP)”, “functional abdominal pain”, “constipation”, and “irritable bowel syndrome”. Randomized controlled trials and randomized crossover trials published in English, with more than fifteen subjects, were included. Only studies involving infants, children, and adolescents (0–21 years of age), with functional gastrointestinal complaints, were included in this review. Studies were included if patients were diagnosed with FC, IBS, RAP or functional abdominal pain. We excluded studies that followed children with organic cause of defecation disorders, such as Hirschsprung’s disease, cystic fibrosis, anorectal malformations, or hypothyroidism. Studies that compared fiber with lactulose, polyethylene glycol, or placebo were included. Abstracts were screened for eligibility, and those that met the criteria were read in full.

## 3. Results

**Figure 1:**
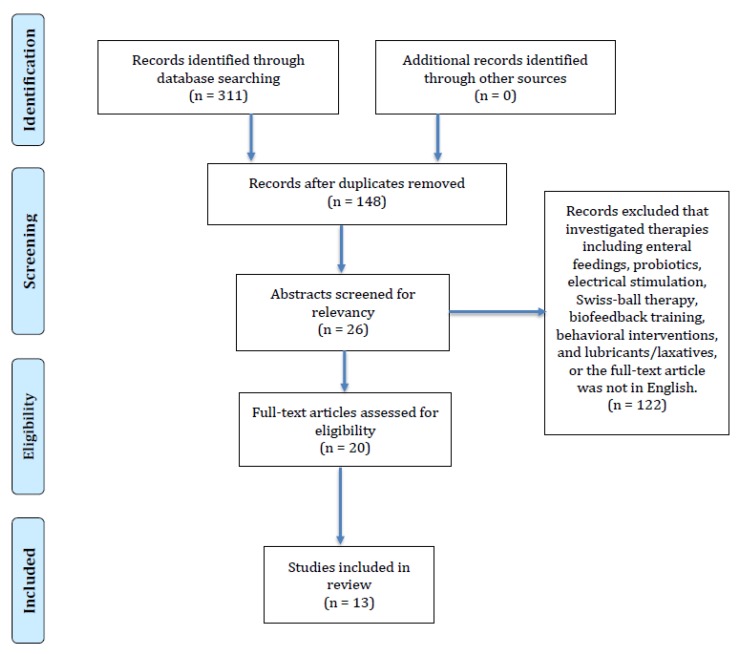
Flow diagram of the available evidence on the role of fiber, in the treatment of functional constipation (FC) and Irritable Bowel Syndrome (IBS) in children.

Three hundred and eleven studies were identified. One hundred and forty-eight were non-duplicates and considered eligible. One hundred and twenty-two studies were excluded for the following reasons: The investigated therapies were enteral feedings, probiotics, electrical stimulation, Swiss-ball therapy, biofeedback training, behavioral interventions, and lubricants/laxatives, or the full-text article was not in English. Twenty-six abstracts were screened for relevancy by two reviewers. A total of twenty articles were reviewed in full by the authors. Finally, thirteen full-text clinical trials with a total of seven hundred and twenty-three pediatric participants met the inclusion criteria and were included in this review. We performed a descriptive analysis on the studies. Figure 1 shows the results of our literature search.

### 3.1 Studies on Single Fiber Types

#### 3.1.1. Corn Fiber

Corn fiber is an insoluble residual endosperm, left after grain processing [21]. Due to its ability to resist digestion in the small intestine, it passes into the large intestine where it is fermented [22]. Corn fiber has been shown to improve laxation, when added to processed foods and beverages, in healthy adults [23].

One early (1985) randomized control trial evaluated corn fiber in children with abdominal pain [24]. Feldman et al. found that supplementing the diet with corn fiber reduced abdominal discomfort [24]. In this study, fifty-two children received either a high fiber cookie or a placebo cookie for six weeks. Primary outcome measures were intensity and frequency of abdominal pain. Each fiber cookie contained 5 g of corn fiber and children were instructed to consume them twice daily. Pain was rated using a “Stomach Ache Diary” four times daily. Pain episodes were significantly less frequent in the corn fiber group than the placebo group. Side effects of gas and diarrhea were minimal in both groups (Table 1).

#### 3.1.2. Partially Hydrolyzed Guar gum (PHGG)

Guar gum is a gel-forming fiber from the seeds of Cyamopsi tetragonolobus (guar plant) [27]. PHGG results from the hydrolysis of guar gum; it differs from guar gum as it is non-gel forming. As a water-soluble fiber, PHGG can be easily added into supplements due to its low viscosity, and odorless and tasteless nature [28]. Research in adults has shown that PHGG accelerates the colonic transit time in patients with chronic constipation and improves the frequency of bowel movements [29]. However, when used in the treatment of FC, among hospitalized patients, 10 g of PHGG did not differ in effects, compared to a 30 g high fiber diet, in terms of defecation frequency, fecal consistency, or the need for laxative drug use [30]. Two randomized control studies have evaluated PHGG, in children with FGID, and found promising results [26,31].

Ustundag et al. found PHGG to be as effective as lactulose for constipation related abdominal pain and stool consistency and to have less side effects [31]. In this prospective controlled study, sixty-eight children were randomized to receive either PHGG or lactulose, for four weeks. Children in the PHGG group were given different doses, depending on age (4–6 years: 3 g/day; 6–12 years: 4 g/day; and 12–16 years: 5 g/day), and were instructed to increase their fluid intake. Children in the lactulose group were given 1 mL/kg/day but were not instructed to increase their fluid intake. Bowel diaries were kept by the patients or parents to collect data regarding stools. Sixty-one patients completed the study, and both stool frequency and consistency improved significantly. However, no statistical difference was noted between the groups. The only side effect documented was increased flatulence in the lactulose group. Thus, this study found that treatment with PHGG and lactulose were equally effective for improving stool consistency (Table 2).

A second investigation found similar results. Romano et al. studied PHGG supplementation in patients with both chronic abdominal pain and IBS [26]. In a randomized, double-blind pilot study, sixty children received either PHGG or placebo for four weeks to determine a change in severity of abdominal pain and IBS. Children in the treatment group were provided 5 g/day of PHGG, mixed in fruit-juice, whereas the placebo group received only fruit-juice. Three validated questionnaires were used to measure clinical symptoms, which were the “Birmingham IBS Symptom Questionnaire”, “Wong Baker Faces Pain Rating Score”, and the “Bristol Stool Scale”. The group treated with PHGG reported greater normalization of bowel movements, reduction in clinical symptoms of IBS and intensity of abdominal pain, compared to the control group (Table 1).

#### 3.1.3. Glucomannan

Glucomannan is a nonabsorbable fiber from the Japanese Konjac plant [41]. It has traditionally been consumed in jelly, noodles, jams, as well as other food products and in Chinese medicine [41]. Clinical studies in healthy adults have demonstrated that Glucomannan improves the colon’s health by increasing stool bulk and stool frequency, without causing gastrointestinal side effects [42]. Three studies evaluated the effect glucomannan supplementation on childhood constipation [32,33,36].

Staiano et al. evaluated the effects of glucomannan on FC in neurologically impaired children [32]. In a double-blind placebo-controlled clinical trial, twenty children with brain damage and chronic constipation received either glucomannan or placebo for twelve weeks to assess the effect of glucomannan, in colonic transit and stool frequency (primary outcomes). Glucomannan was administered at 100 mg/kg twice daily. Bowel movements, consistency, and presence of abdominal pain were recorded daily, via a diary card. Nineteen participants completed the treatment phase. The authors found that treatment with glucomannan significantly increased stool frequency and decreased consistency and painful defecation, but there were no significant effects on colonic transit (Table 2).

Loening-Baucke et al. found that glucomannan improves stool frequency [33]. In a double-blind, randomized, crossover study, thirty-one children received either fiber or placebo for four weeks. Glucomannan was dosed at 100 mg/kg of body weight, daily, with a maximum dose of 5 g per day. Abdominal pain and stools were tracked using a diary that evaluated daily bowel movements and episodes of fecal incontinence and pain. Glucomannan treatment was considered successful if children experienced greater or equal to three bowel movements per week and less than one fecal incontinence episode, in the previous three weeks. The authors found that more children experienced a significantly more success with the fiber treatment than with placebo (Table 2).

Chmielewska’s findings contradict the results of the previous two studies [36]. In a double-blind, randomized placebo-controlled trial, eighty children received either glucomannan or placebo for four weeks. Treatment was successful if there were ≥3 stools per week and no episodes of fecal incontinence. Glucomannan was dosed at 2.52 g per day. Bowel diaries were used to document stool patterns. Seventy-two participants completed the study, with twenty children in the glucomannan group experiencing success, compared to twenty-one children in the placebo group. Thus, the study did not find a significant difference in therapeutic success between glucomannan and placebo, for FC in children (Table 2).

#### 3.1.4. Inulin

Inulin is a polysaccharide commonly found in roots, tubers, and plant rhizomes from common foods, including asparagus, chicory, garlic, onion, and artichoke [43]. A 2014 meta-analysis [44] of randomized controlled clinical trials involving two hundred and fifty-two subjects (four adult studies and one pediatric study that was included in this review) found that inulin improved stool frequency, stool consistency, and transit time. Since the publication of the meta-analysis, one more study has evaluated the effect of inulin on children with constipation [40].

In 2017, Closa-Monasterolo et al. assessed the effects of inulin-type fructans in children with FC [40]. In a double-blind, randomized, placebo-controlled parallel group trial, twenty-two children received either two doses of inulin-type fructans or placebo for six weeks to determine its effect in stool consistency and frequency. The inulin-type fructans were provided at 4 g daily. Seventeen children completed the study. It was found that children who received the inulin-type fructans had decreased stool consistency, according to the modified Bristol Scale (Table 2).

#### 3.1.5. Oligosaccharides

Oligosaccharides are a non-digestible complex carbohydrate source [45] that increases fecal weight and water in the stools [46]. Galactooligosaccharides (FOS) exemplify this type of soluble dietary fiber [39]. One study evaluated the effect of oligosaccharides on stool consistency in the pediatric population [39].

Beleli et al. assessed the effects of galactooligosaccharides (GOS) on constipated patients and found that it improved the GI symptoms [39]. In a double-blinded, placebo-controlled, crossover trial, twenty patients received either GOS or placebo for thirty days to evaluate changes in constipation signs and symptoms. A fifteen-day washout period separated the trial, followed by another thirty days on the alternative intervention. GOS were consumed at 1.7 g, daily, and home visits occurred, biweekly, to collect data and measure compliance and symptoms. GOS ingestion resulted in significant improvements, compared to placebo, including bowel movement frequency, relief of straining during defecation, and a decrease in stool consistency. No side effects were noted (Table 2).

#### 3.1.6. Cocoa husk

The outer shell of cocoa beans that encase the nibs are rich in soluble fiber [47]. One study evaluated the effect of Cocoa husk supplementation on childhood FC.

Castillejo et al. found that supplementation with cocoa husk improved the intestinal transit time, as well as reduced the percentage of patients who reported hard stools [34]. In a parallel, randomized, double-blind, controlled trial, fifty-six children received either a cocoa husk supplement or placebo for four weeks, in addition to the toilet regimen. Total colonic transit time was the primary outcome of treatment success. The patient’s parents assessed the bowel movement and stool consistency, via a diary. Forty-eight children completed the study, with a significant decrease in the intestinal transit time, in the cocoa husk group, from 45.4 ± 38.4 h, versus 8.7 ± 28.9 h, in the placebo group. There was a significant difference in stool consistency in favor of cocoa husk. The cocoa husk group also noted an improvement in the frequency of bowel movements but the difference was non-significant. There were no side effects reported during the study (Table 2).

#### 3.1.7. Psyllium fiber

Psyllium is a common over the counter fiber supplement. Psyllium is derived from the seeds of the plant genus Plantago, and acts as soluble viscous fiber [48]. Due to its ability to hold water, it is sometimes used to treat both constipation and mild diarrhea. One randomized control study evaluated psyllium in children with constipation [25].

Shulman et al. found that the psyllium fiber lessened the frequency of abdominal pain in children with IBS [25]. In a randomized, double-blind trial, eighty-four children received either psyllium or placebo for six weeks. Children 7–11 years of age, received 6 g of psyllium per day, while those of age 12–18 years, received 12 g. Participants recorded the daily abdominal pain severity and pain frequency. Interestingly, those treated with psyllium experienced a significant reduction of pain episodes but not pain intensity (Table 1).

### 3.2 Studies on Combined Fiber Types

#### Transgalacto-oligosaccharides, inulin, soy fiber, resistant starch, acacia fiber, and psyllium fiber

It has been speculated that a combination of different fiber types, including short and long chain fibers may be a more effective approach for controlling constipation, due to its action on different parts of the colon. Three studies evaluated the effects of a combination of fiber blends, in children with constipation [35,37,38].

A study hypothesized that a combination of fibers would have a more beneficial effect, compared to lactulose, which acts mainly in the proximal colon. However, the hypothesis was unproven. Kokke et al. [35] did not find a fiber mixture to be a more effective for the treatment of childhood constipation. In a randomized, double-blind, prospective controlled study, one hundred and thirty-five participants received either a yogurt drink with mixed dietary fibers or lactulose for 8 weeks. Children were then weaned for 4 weeks. The fiber mixture contained 3.0 g transgalacto-oligosaccharides, 3.0 g inulin, 1.6 g soy fiber, and 0.33 g resistant starch, per 100 mL. Parents, or the patient, recorded the defecation frequency, consistency of stool, abdominal pain, flatulence, and episodes of fecal incontinence, in a bowel diary. Ninety-seven children completed the investigation (28% drop out rate). The authors did not find a difference in defection frequency or fecal incontinence between both groups. Those treated with lactulose noted softer stools. Thus, lactulose and a dietary fiber mixture were found to be equally effective for the treatment of childhood constipation (Table 2).

Data suggests gel-forming fibers soften hard stools in constipation; although fermented in different areas of the bowel, Acacia fiber and psyllium are two examples of the gel-forming fibers [49]. One randomized control study evaluated the effects of these fiber blends in children with constipation [37].

Quitadamo et al. studied a fiber mixture, acacia fiber, psyllium fiber, and fructose, and found that the fiber mixture improved constipation comparable to polyethylene glycol (PEG) [37]. In a randomized, open-label, prospective, controlled, parallel-group study, hundred children received either the fiber mixture or PEG, for eight weeks, to determine effects on the gastrointestinal symptoms. For eight weeks, children were provided with 16.8 g of the fiber mixture. Gastrointestinal symptoms were recorded via stool diary. The frequency of bowel movements and stool consistency were improved in both groups, although PEG was deemed more palatable (Table 2).

Weber et al. evaluated a fiber mix in the treatment of chronic constipation in children [38]. In a placebo-controlled, double-blind study, fifty-seven children received either a fiber mixture of several prebiotic sources, including FOS, inulin, gum acacia, resistant starch, soy polysaccharides, and cellulose, or placebo for four weeks. Dosing was based on the child’s body weight. Children under 18 kg received 3.8 g of fiber, twice daily, and was compared to children >18 kg that received 7.6 g of fiber twice daily. Participants completed the daily stool questionnaires. Patients in the fiber group significantly increased the frequency of bowel movements, as well as improved their stool consistency (Table 2).

### 3.3. Children’s Acceptance of Fiber

As important as fiber supplementation, is its acceptability by children. Two studies investigated whether children are amenable to increase their dietary fiber intake. Brauchla et al. [50] studied the effect of introducing two high fiber snacks per day. In a randomized controlled prospective intervention study, children between 7 and 11 years were given either two high fiber snacks per day, or their usual snacks for eight weeks. Twenty-four-hour dietary recalls were used to assess intake and acceptability. It was found that children who were offered the high fiber snacks increased their fiber intake by 2.5 g per day. Making high fiber foods and snacks more consistently available to children is suggested as a means of increasing fiber intake.

Long-term adherence to a high fiber diet can also be improved with a team approach, including a physician and registered dietitian. Karagiozoglou-Lampoudi et al. [51] studied the compliance to a high fiber/high water regime in children with a refractory FC. In a prospective randomized study, forty-two children followed their physician’s dietary advice for the management of constipation, while forty-four children received dietary management by a registered dietitian. Children seen by a dietitian increased their fiber and water intake more than those who were not. Personalized dietary modification by a nutrition professional is recommended to optimize children’s compliance to high fiber/high water diet.

## 4. Discussion

Children consume a suboptimal amount of dietary fiber, due to limited intake of vegetables, fruits, and whole grains [52]. In 2008, the Feeding Infants and Toddlers Study (FITS) assessed the usual nutrient intakes of three thousand two hundred and seventy-three infants, toddlers, and preschoolers. Through a random sample of US children, it was determined that most toddlers and preschoolers consume a low-intake of dietary fiber [52]. Anecdotal evidence suggests that children who do not consume enough fiber experience more constipation, but the role of supplemental fiber in treating FC, remains controversial [53].

Fibers are also known to influence microbiota composition. The gut contains over a 100 trillion microbial cells, which have an impact on physiology, metabolism, and nutrition [54]. Inadequate fiber intake in the long-term may contribute to a gut dysfunction by reducing these important microbial taxa. Increasing fiber intake slowly, over time, is suggested as it will allow the microbiota to adapt to higher doses [54].

Still, the NASPGHAN/ESPGHAN guidelines published in 2014 [10] stated that there was no evidence to support the use of fiber in the treatment of FC in children, and therefore recommend giving children a “normal” amount of fiber for the child’s age. The Rome IV criteria, published in 2016 [1], does not mention the use of fiber among the recommended treatments for IBS in children. Since then, three pediatric randomized clinical trials have been published, two of them on the treatment of FC and one on IBS. Although all of them showed positive results, we found great variability among the studies we reviewed. Thus, despite most of them having positive results, we believe that previous recommendations continue to be valid.

Our review included ten randomized clinical trials on the efficacy of fiber, for the treatment of FC. The studies differed in methods, fiber types, doses, endpoints, and age groups. Among these studies, some had a placebo arm as comparator and others compared fiber against other treatments. The variability among studies in combination with the small size of some of them does not allow combining the data for a meta-analysis.

There are at least twenty-six different types of fibers. Out of all types of fiber, only nine types have been studied for the treatment of FC and IBS in children (guar gum, glucomannan, cocoa husk, psyllium, inulin, corn fiber, acacia fiber, oligosaccharides, and soy fiber). One of the fibers most commonly studied is glucomannan. Two, out of three small studies, [32,33,36] concluded that its use was beneficial for the treatment of FC.

In one of these studies, children received glucomannan, in combination with a standard laxative treatment, while another trial was conducted in children with FC and the altered neurological status. Both studies showed significant benefits. A third study [36] did not find glucomannan to be effective; however, the dose of glucomannan used in this study was low. Although the results of the trials of glucomannan could be considered encouraging, more studies should be conducted before recommending its use in clinical practice.

Two studies [31,35] examined the use of dietary fiber versus lactulose, in the treatment of FC, using a different design. Both studies concluded that they were equally effective. None of the studies included a placebo arm. The design of Ustundag’s study made it prone to bias. Only the subjects treated with PHGG were recommended to increase their fluid intake, while children in the placebo group were not. Although there is no evidence that fluid is beneficial for the treatment of FC, we cannot rule out that the design of the study influenced the outcome. The dropout rate in Kokke’s study was unusually high.

Differentiating between the IBS-C and FC is sometimes challenging [55]. Forty-five percent of children with FC report abdominal pain [55] and it is sometimes unclear if some of them, in fact, may have IBS-C. Poorly absorbed oligosaccharides are FODMAPs that can trigger IBS symptoms. Interestingly, three studies assessed the effect of FODMAP fibers for the treatment of FC. Closa-Monasterolo and Kokke found that inulin supplementation improved stool consistency, in children with FC. These results parallel the findings with other oligosaccharides as well [39]. Chumpitazi et al. [56] found that fructans exacerbate the IBS symptoms in children. None of the studies [35,39,40] reported any side effects resulting from the use of FODMAPs, for the treatment of FC. This may indicate that the diagnosis of FC can be well differentiated at the time of the inclusion criteria or that the lower dose of FODMAPs used in these studies was not enough to trigger the detrimental symptoms.

Only three randomized clinical trials with a total of a hundred and ninety-six children were published in English, in the last thirty-three years, for the treatment of IBS. All of them used a placebo arm and showed positive results, although the study design and type of fiber differed. Romano et al. studied the effect of PHGG in children with IBS and chronic abdominal pain. Shulman et al. studied the effect of psyllium in IBS and Feldman studied the effect of corn diet in children with RAP. The differences in inclusion criteria, design, and endpoints did not allow us to arrive at a firm conclusion on the effect of fiber, in children with IBS.

One objective of the 2010 Dietary Guidelines [57] was to increase the dietary fiber (DF) intake. None of the studies investigated the effects of increasing fiber from whole foods rich in fiber that could be a more “natural” way of increasing the fiber intake. Fiber from whole foods may yield different results than that of supplemental fiber therapy. Thus, studies that evaluate the impact of increasing fiber, from whole foods, in children with FC and IBS, should be encouraged.

To improve adherence and therapeutic success, recommendations for treatment need to be simple and easy to understand. Three studies [35,37,38] utilized a combination of multiple fiber types in their treatment, which may be difficult to replicate naturally, in daily practice. Although the studies found their treatments to be comparable to PEG and lactulose, it is well established that parents’ adherence to physician recommendations is low [18], and advising multiple fiber types may be impracticable.

There are limitations to this review. Our review only included studies in English. Some positive trials in other languages may have not been considered. We cannot exclude that our review would have arrived at a different conclusion if studies in other languages had been included. One of the studies used fiber in low doses [36]. It is unknown whether fiber used at a larger dose could have resulted in better outcomes. However, the use of high doses of fiber may not always be beneficial. Bulkier stools may be uncomfortable for some children and a diet rich of some types of fibers (FODMAPs) may be detrimental.

## 5. Conclusions

Most studies on the use of fiber for the treatment of FC and IBS have shown its benefit. However, due to differences in design, such as the use of multiple types of fibers, dosing and length of treatment and small sample sizes and risk of bias, we are unable to make a definitive recommendation supporting the use of fiber for the treatment of FC and IBS. The conclusions of our review are, thus, in agreement with NASPHGAN/ESPGHAN recommendations that a normal fiber intake should be encouraged in children with and without FC. The small number of clinical trials on the use of fiber in children with IBS do not allow for arriving at a definitive conclusion. Practitioners should be cautious at the time of selecting the type and dose of fibers in children with IBS to avoid worsening symptoms.

## Figures and Tables

**Table 1:** Clinical Trials on Dietary Fiber for the Management of Irritable Bowel Syndrome.

Author	Year Published	Location	Type of Study	*N*	Age (Years)	Fiber Type	Soluble or Insoluble	Primary Outcome	Secondary Outcome	Dose	Length of Treatment	Results
**William Feldman et al. [24]**	1985	Canada	Randomized, double-blind, parallel trial	52 Per intention to treat analysis	5–15	Corn fiber vs. placebo	Soluble	50% decrease in frequency of pain episodes	None listed	10 g daily	2 weeks	*p* = 0.04
**Robert Shulman et al. [25]**	2017	USA	Randomized, double-blind parallel trial	84 Per protocol analysis	7–18	Psyllium vs. placebo	Soluble	Pain episodes Pain severity Percentage of normal stools	Changes in breath hydrogen/methane production Gut permeability	7–11 years: 6 g daily 12–18 years: 12 g daily	6 weeks	Pain episodes: *p* = 0.03 Pain severity: *p* = 0.97 Percentage of normal stools: *p* = 0.32 Breath hydrogen: *p* = 0.11/ Breath methane: *p* = 0.51 Gut permeability: NS
**Claudio Romano et al. [26]**	2013	Italy	Randomized, double-blind parallel trial	60 Per intention to treat analysis	8–16	Partially hydrolyzed guar gum vs. placebo	Soluble	Stool frequency Intensity of clinical symptoms	Compliance	5 g daily	4 weeks	Stool frequency: *p* = 0.025 Intensity: *p* > 0.05

**Table 2:** Clinical Trials on Dietary Fiber for the Management of Functional Constipation.

Author	Year Published	Location	Type of Study	*N*	Age (Years)	Fiber Type	Water Soluble or Insoluble	Primary Outcome	Secondary Outcome	Dose	Length of Treatment	Results
**Annamaria Staiano et al. [32]**	2000	Italy	Randomized, double-blind parallel trial	19 Per protocol analysis	5.7 ± 4.2	Glucomannan vs. placebo	Soluble	Stool frequency Reduction of laxative or suppository use Stool consistency Painful defecation Colonic transit time	None listed	100 mg/kg twice daily	12 weeks	Stool frequency: *p* < 0.01 Reduction of laxative or suppository use: *p* < 0.01 Stool consistency: *p* < 0.01 Painful defecation: *p* < 0.01 Colonic transit time: *p* < 0.05
**Vera Loening-Baucke et al. [33]**	2004	USA	Randomized, double-blind crossover trial	31 Per protocol analysis	4.5–11.7	Glucomannan vs. placebo	Soluble	Successful treatment: (≥3 BMs per week and ≤1 soiling episode in the last 3 weeks with no abdominal pain)	None listed	100 mg/kg body weight daily Maximum dose: 5 g daily	4 weeks	Successful treatment: *p* < 0.05
**Gemma Castillejo et al. [34]**	2006	Spain	Randomized, double-blind parallel trial	56 Per protocol analysis	3–10	Cocoa husk vs. placebo	Insoluble	Colonic transit time	Stool frequency Stool consistency Pain improvement	3–6 years: 8 g daily 7–10 years: 16 g daily	4 weeks	Colonic transit time: *p* < 0.015 Stool frequency: *p* = 0.780 Stool consistency: *p* = 0.039 Pain improvement: *p* = 0.109
**Freddy T.M. Kokke et al. [35]**	2008	The Netherlands	Randomized, double-blind parallel trial	97 Per protocol analysis	1–13	Transgalacto-oligosaccharides + Inulin + Soy fiber + Resistant Starch vs. lactulose	Soluble (Transgalacto-oligosaccharides, Inulin, Resistant Starch) Insoluble (Soy fiber)	Stool frequency	Fecal incontinence Stool consistency Abdominal painFlatulence	<15 kg: 10 g daily 15–20 kg: 20 g daily >20 kg: 30 g daily	8 weeks	Stool frequency: *p* = 0.481 Fecal incontinence: *p* = 0.084 Consistency: *p* = 0.01 Abdominal pain: *p* = 0.395 Flatulence: *p* = 0.739
**Gonca Ustundag et al. [31]**	2010	Turkey	Randomized, prospective parallel trial	61 Per protocol analysis	4–16	Partially hydrolyzed guar gum vs. lactulose	Soluble	Stool frequency Stool consistency Abdominal pain	None listed	4–6 years: 3 g daily 6–12 years: 4 g daily 12–16 years: 5 g daily	4 weeks	Stool frequency: *p* <0.05 Stool consistency: *p* <0.05 Abdominal pain: *p* <0.05
**Anna Chmielewska et al. [36]**	2011	Poland	Randomized, double-blind parallel trial	72 Per protocol analysis	3–16	Glucomannan vs. placebo	Soluble	Treatment success: (>3 stools per week with no soiling)	Stool consistency Stool frequency Episodes of fecal soiling Episodes of painful defecation Episodes of flatulence Episodes of abdominal pain	2.52 g daily	4 weeks	Treatment success: *p* > 0.99 Stool consistency: *p* = 0.58 Stool frequency: *p* = 0.11 Fecal soiling: *p* = 0.08 Painful defecation: *p* = 0.42 Flatulence: *p* = 0.78 Abdominal pain: *p* <0.0001
**Paolo Quitadamo et al. [37]**	2012	Italy	Randomized, prospective, open label, parallel trial	100 Per protocol analysis	4–10	Acacia fiber + Psyllium fiber + fructose vs. polyethylene glycol 3350 with electrolytes	Soluble (acacia fiber, psyllium fiber)	Stool frequency Stool consistency Fecal incontinence	Improvement of gastrointestinal symptoms	16.8 g daily	8 weeks	Stool frequency: *p* = 0.621 Stool consistency: *p* = 0.751 Fecal incontinence: *p* = 0.621
**Thabata K. Weber et al. [38]**	2014	Brazil	Randomized, double-blind parallel trial	54 Per protocol analysis	4–12	Fructooligosaccharides, inulin, gum arabic, resistant starch, soy polysaccharide, cellulose vs. placebo	Soluble (fructooligosaccharides, inulin) Insoluble (cellulose)	Therapeutic failure (oral stool softeners or enemas was required during the trial)	Stool frequency Stool consistency	<18 kg: 7.6 g daily >18 kg: 15.2 g daily	4 weeks	Therapeutic failure: *p* = 0.933 Stool frequency: *p* = 0.014 Stool Consistency *p* = 0.003
**Célia A.V. Beleli et al. [39]**	2015	Brazil	Randomized, double-blind crossover trial	20 Per protocol analysis	4–16	Galactooligosaccharides vs. placebo	Soluble	Stool frequency Straining Stool consistency	None listed	1.7 g daily	30 days	Stool frequency: *p* < 0.0001 Straining: *p* < 0.0001 Stool consistency: *p* = 0.0014
**Ricardo Closa-Monasterolo et al. [40]**	2017	Spain	Randomized, double-blind parallel trial	17 Per protocol analysis	2–5	Inulin-type fructans vs. placebo	Soluble	Stool consistency	Stool frequency Gastrointestinal symptoms	4 g daily	6 weeks	Stool consistency: *p* = 0.040 Stool frequency: *p* = 0.328 Gastrointestinal symptoms (pain): *p* = 0 .014
